# Glycoprotein B Cleavage Is Important for Murid Herpesvirus 4 To Infect Myeloid Cells

**DOI:** 10.1128/JVI.00709-13

**Published:** 2013-10

**Authors:** Daniel L. Glauser, Ricardo Milho, Bruno Frederico, Janet S. May, Anne-Sophie Kratz, Laurent Gillet, Philip G. Stevenson

**Affiliations:** Division of Virology, Department of Pathology, University of Cambridge, Cambridge, United Kingdoma; Institute of Virology, Vetsuisse Faculty, University of Zurich, Zurich, Switzerlandb

## Abstract

Glycoprotein B (gB) is a conserved herpesvirus virion component implicated in membrane fusion. As with many—but not all—herpesviruses, the gB of murid herpesvirus 4 (MuHV-4) is cleaved into disulfide-linked subunits, apparently by furin. Preventing gB cleavage for some herpesviruses causes minor infection deficits *in vitro*, but what the cleavage contributes to host colonization has been unclear. To address this, we mutated the furin cleavage site (R-R-K-R) of the MuHV-4 gB. Abolishing gB cleavage did not affect its expression levels, glycosylation, or antigenic conformation. *In vitro*, mutant viruses entered fibroblasts and epithelial cells normally but had a significant entry deficit in myeloid cells such as macrophages and bone marrow-derived dendritic cells. The deficit in myeloid cells was not due to reduced virion binding or endocytosis, suggesting that gB cleavage promotes infection at a postendocytic entry step, presumably viral membrane fusion. *In vivo*, viruses lacking gB cleavage showed reduced lytic spread in the lungs. Alveolar epithelial cell infection was normal, but alveolar macrophage infection was significantly reduced. Normal long-term latency in lymphoid tissue was established nonetheless.

## INTRODUCTION

Glycoprotein B (gB) is a conserved herpesvirus virion component involved in cell binding and viral membrane fusion (1). It and the structurally homologous vesicular stomatitis virus (VSV) G protein and baculovirus gp64 are considered to be class III fusion proteins (2–7). Most herpesvirus gBs contain an R-X-K/R-R recognition motif for the ubiquitously expressed, subtilisin-like cellular endoprotease furin (8). Furin has many substrates, including cellular proteins, bacterial toxins, and glycoproteins from avian influenza virus, human immunodeficiency virus, measles virus, respiratory syncytial virus, and Ebola virus (9). It acts in the *trans*-Golgi network (TGN), plasma membrane, and early endosomes (9), and gB cleavage is presumed to occur in the TGN (10).

The role of gB furin cleavage has been addressed for several herpesviruses. For pseudorabies virus (PRV), cleavage of gB by furin is not required for cell entry or replication *in vitro* but is important for syncytium formation (11); mutation of the furin cleavage site (FCS) of bovine herpesvirus 1 does not affect viral replication but reduces plaque size, consistent with impaired cell-to-cell spread (12); varicella-zoster virus FCS mutation affects neither replication kinetics nor plaque size *in vitro* but significantly impairs replication in human skin xenografts in SCID mice (13); human cytomegalovirus (HCMV) FCS mutation does not affect replication *in vitro* (14); and Epstein-Barr virus (EBV) FCS mutation reduces transfection-based cell-to-cell fusion (15). Together, these studies argue that gB cleavage is nonessential for herpesvirus replication *in vitro* but may have a role in gB fusion activity that manifests as reduced cell-to-cell spread. However, none of these analyses addressed directly the role of gB cleavage in host colonization, that is, entry, primary lytic replication, dissemination, and latency establishment. To do this, we employed the accessible, small-animal gammaherpesvirus infection model provided by murid herpesvirus 4 (MuHV-4).

MuHV-4 is genetically closer to Kaposi's sarcoma-associated herpesvirus (KSHV) than to EBV (16) but shares with EBV an infectious mononucleosis syndrome, exploitation of host germinal centers, and long-term latency in memory B cells (17, 18). Thus, it is likely that all these viruses colonize their hosts in fundamentally similar ways. Upon intranasal inoculation, MuHV-4 replicates in the respiratory tract and is trafficked by dendritic cells (DCs) to the draining lymph nodes, where it is transmitted to B cells (19–21). B cell activation and proliferation then drive a T cell mononucleosis (21–25).

The gB of MuHV-4 is predicted to have a structure very similar to those of herpes simplex virus 1 and EBV. MuHV-4 membrane fusion is blocked by gB-specific monoclonal antibodies (MAbs) that recognize an epitope close to one of the predicted fusion loops (26). Like EBV and KSHV, the MuHV-4 gB contains a furin recognition motif, and the 120-kDa precursor protein is cleaved into 65- and 55-kDa disulfide-linked N- and C-terminal fragments (27). Cell-associated gB is predominantly uncleaved while virion gB is almost entirely cleaved, suggesting that cleavage occurs during gB incorporation into virions (27). This would be consistent with the TGN being one of the main localizations of both furin activity and herpesvirus secondary envelopment (9, 10, 28).

We prevented MuHV-4 gB cleavage by mutating its FCS. *In vitro*, the mutant viruses entered fibroblasts and epithelial cells normally but entered myeloid cells such as macrophages and DCs poorly. After intranasal infection of mice, viruses lacking gB cleavage showed significantly less lytic spread in the lungs. Histological analysis showed reduced infection of alveolar macrophages, while alveolar epithelial cell infection was normal. Thus, MuHV-4 gB cleavage is important for myeloid cell infection both *in vitro* and *in vivo*.

## MATERIALS AND METHODS

### Cells.

BHK-21, NMuMG, NS0, and RAW 264.7 cells (all from ATCC) were grown in Dulbecco's modified Eagle's medium (DMEM; high glucose; PAA Laboratories) with 2 mM l-glutamine (PAA Laboratories), 100 U/ml penicillin and 100 μg/ml streptomycin (PAA Laboratories), and 10% fetal bovine serum (FBS; PAA Laboratories or Amimed) (complete medium). Peritoneal cavity cells were isolated from BALB/c mice by peritoneal lavage and cultivated in RPMI 1640 (PAA Laboratories) with 10% heat-inactivated (56°C, 30 min) FBS (PAA Laboratories or Amimed), 2 mM l-glutamine (PAA Laboratories), and 50 U/ml penicillin and 50 μg/ml streptomycin (PAA Laboratories). For identification of F4/80^high^ peritoneal macrophages by flow cytometry, immunoglobulin Fc receptors (FcRs) were blocked (15 min, 4°C) with anti-mouse CD16/32 MAb (clone 2.4G2; BD Biosciences) diluted 1:100 in phosphate-buffered saline (PBS) containing 4% FBS and the cells were stained (1 h, 4°C) with anti-mouse F4/80-allophycocyanin (APC) conjugate (clone BM8; BioLegend) diluted 1:200 in PBS containing 4% FBS. DCs were grown from bone marrow progenitors of BALB/c mice in RPMI 1640 (PAA Laboratories) with 10% heat-inactivated (56°C, 30 min) FBS (PAA Laboratories or Amimed), 2 mM l-glutamine (PAA Laboratories), 50 μM 2-mercaptoethanol (Invitrogen), 100 U/ml penicillin and 100 μg/ml streptomycin (PAA Laboratories), and 8 ng/ml granulocyte-macrophage colony-stimulating factor (GM-CSF; Invitrogen). Bone marrow cells were first plated onto tissue culture plastic (30 min, 37°C), and the adherent (macrophage-rich) cells were discarded. After 3 days of culture of the remaining cells, the nonadherent (granulocyte-rich) cells were removed by washing and the adherent cells were supplemented with fresh medium. After 5 days, the cells were again supplemented with fresh medium. After 7 days, both the adherent and nonadherent cells were harvested. For identification of CD11c^high^ DCs by flow cytometry, FcRs were blocked (15 min, 4°C) with anti-mouse CD16/32 MAb (clone 2.4G2; BD Biosciences) diluted 1:100 in PBS containing 4% FBS and the cells were stained (1 h, 4°C) with anti-mouse CD11c-APC conjugate (clone N418; BioLegend) diluted 1:200 in PBS containing 4% FBS.

### Mice.

Female BALB/c mice were purchased from Harlan and housed in the Cambridge University Department of Pathology. Mice were infected with MuHV-4 at the age of 6 to 8 weeks by intranasal inoculation in 30 μl complete medium/mouse under general anesthesia with isoflurane or by intraperitoneal injection. All regulated procedures were performed under Home Office Project License 80/1992.

### Viruses.

**Fig 1:**
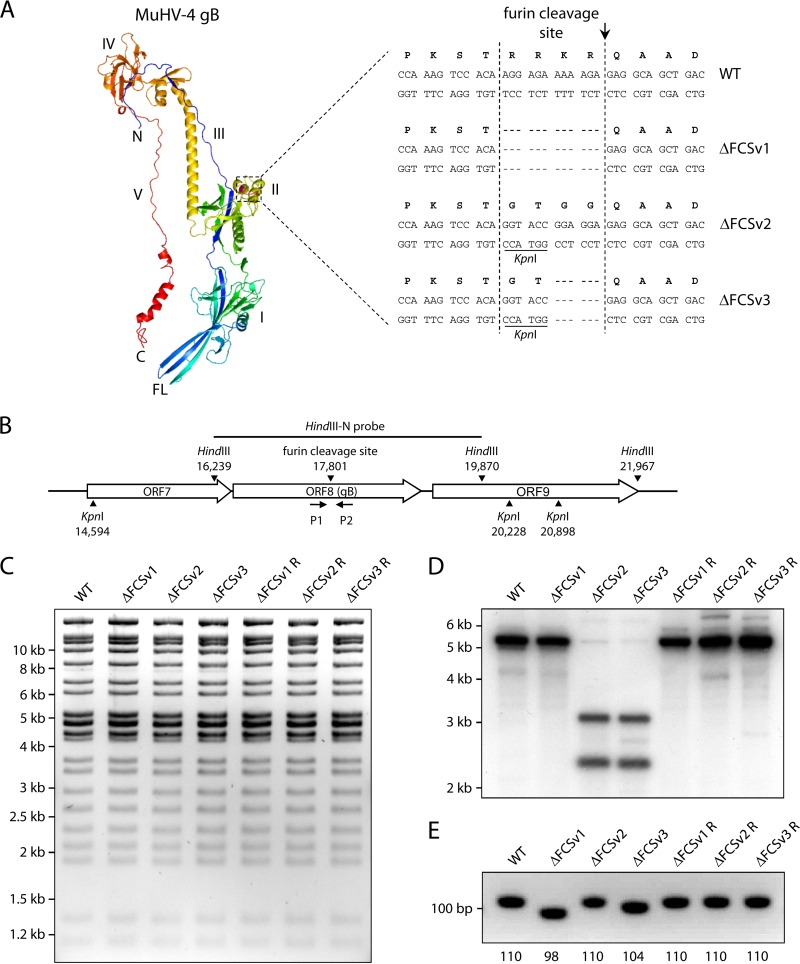
Mutation of the furin cleavage site (FCS) of MuHV-4 gB. (A) The location of the FCS within a structure model of the MuHV-4 gB ectodomain (amino acids 60 to 680) is shown in purple. The amino acid and nucleotide sequences of the wild-type (WT) FCS and the ΔFCSv1, ΔFCSv2, and ΔFCSv3 mutants are shown. A diagnostic KpnI restriction site was introduced in the ΔFCSv2 and ΔFCSv3 mutants. The site at which furin is expected to cleave gB is indicated by an arrow. I to V, domains I to V; N, amino terminus; C, carboxy terminus; FL, fusion loops. (B) Schematic drawing of the MuHV-4 ORF8 encoding gB. HindIII and KpnI restriction sites, the HindIII fragment used as probe for Southern blot hybridization, and the position of primers P1 and P2 used for PCR analysis are shown. (C) Restriction enzyme analysis of mutant MuHV-4 BACs. The WT BAC and ΔFCSv1, ΔFCSv2, and ΔFCSv3 mutant and revertant BACs were digested with HindIII and separated by agarose gel electrophoresis. (D) Southern blot analysis of mutant viruses. Viral DNA was extracted, digested with KpnI, and analyzed by Southern blotting hybridization with the radioactively labeled MuHV-4 HindIII N fragment. In the mutant viruses ΔFCSv2 and ΔFCSv3, the 5.6-kb KpnI fragment is cleaved into 3.2-kb and 2.4-kb bands due to the introduced KpnI site. (E) PCR analysis of mutant viruses. Viral DNA was subjected to PCR with primers P1 and P2 spanning the sequence encoding the FCS, followed by agarose gel electrophoresis. The observed PCR products correspond to the expected sizes shown below the gel image (in bp).

All viruses used in this study were derived from the previously described bacterial artificial chromosome (BAC) clone of MuHV-4 strain 68 (pHA3) (29). The parental M3-LUC MuHV-4 expressing firefly luciferase from the MuHV-4 M3 promoter from an ORF57-ORF58 intergenic expression cassette was described previously (20). The mutations of the gB FCS shown in Fig. 1A were introduced by overlap PCR mutagenesis into plasmid pBrad-gB expressing the gB extracellular domain linked to a glycosylphosphatidylinositol (GPI) anchor (27). The mutations were confirmed by DNA sequencing. The mutant gB sequences were then introduced into the M3-LUC MuHV-4 BAC by RecA-mediated homologous recombination in Escherichia coli as described previously (29, 30). The ΔFCSv2 and ΔFCSv3 mutant BACs were identified by the presence of the newly introduced KpnI site by restriction enzyme digestion and agarose gel electrophoresis. ΔFCSv1 mutant BACs were identified by a diagnostic PCR amplifying a short fragment spanning the gB FCS sequence (genomic coordinates 17747 to 17856) with primers P1 (AGACGAGGACAGCGACCCAG) and P2 (CTGAGCCCTTTGAGACCTCA) using *Taq* DNA polymerase (Invitrogen). The amplicon size was 110 bp for the wild-type (WT) and ΔFCSv2 sequence, 98 bp for the ΔFCSv1 sequence, and 104 bp for the ΔFCSv3 sequence. The overall integrity of the mutant and revertant BACs was verified by EcoRI and HindIII digestion and agarose gel electrophoresis. Mutant viruses were reconstituted by transfection of mutant BACs into BHK-21 cells using FuGENE 6 transfection reagent (Roche). To ascertain that the mutant viruses carried the correct mutations, DNA was extracted from virion preparations using the Wizard genomic DNA purification kit (Promega) and analyzed with the diagnostic PCR described above and by Southern blotting. For the latter, viral DNA was digested with KpnI (Roche), separated on a 1% agarose gel, transferred and UV-cross-linked to a positively charged nylon membrane (Roche), hybridized with a P32-labeled probe consisting of the MuHV-4 genomic HindIII N fragment (genomic coordinates 16239 to 19870), and exposed to X-ray films (Fujifilm). The BAC^+^ viruses retaining the BAC cassette and the HCMV IE1 enhancer/promoter-driven enhanced green fluorescent protein (eGFP) expression cassette therein were used as eGFP^+^ reporter viruses. In order to obtain BAC^−^ viruses for *in vivo* infections, the loxP-flanked BAC cassette was removed from viral genomes by passage through NIH 3T3-Cre cells (31). MuHV-4 stocks were grown in BHK-21 cells (31). The medium of infected cells was harvested, cell debris was removed by low-speed centrifugation (1,000 × *g*, 10 min), and virions were recovered from supernatants by high-speed centrifugation (38,000 × *g*, 90 min). After resuspension of virions in complete medium, potential remaining cell debris and virion aggregates were removed by filtration through a 0.45-μm cellulose acetate filter (Whatman). To determine eGFP titers of eGFP^+^ virus stocks, viruses were added to BHK-21 cells and incubated overnight in the presence of 100 μg/ml phosphonoacetic acid (PAA) to prevent secondary spread, followed by counting the proportion of eGFP^+^ cells with a FACSCalibur (BD Biosciences) or Gallios (Beckman Coulter) flow cytometer and calculation of the multiplicity of infection (MOI) using the Poisson distribution. To determine PFU titers, virus stocks were titrated by plaque assay on BHK-21 cells as described previously (31).

### Antibodies.

The following MAbs derived from MuHV-4 infected BALB/c mice were used: MG-2C10 recognizing a linear epitope in gB N terminal of the FCS (32), MG-4D11 recognizing a linear epitope in gB C terminal of the FCS (32), BN-1A7 recognizing a conformational epitope in prefusion gB (33), SC-9E8 and SC-9A5 recognizing neutralizing conformational epitopes in prefusion gB (26), MG-1A12 recognizing a conformational epitope in postfusion gB (33), 3F7 recognizing a linear epitope in gN (34), T2C12 and 8F10 recognizing neutralizing conformational epitopes in gH complexed with gL (gH/gL) (35), and BN-3A4 and T1A1 recognizing linear epitopes in gp150 (36). Staining of cells for flow cytometry and enzyme-linked immunosorbent assay (ELISA) was done with hybridoma supernatants, while antibody stocks concentrated by ammonium sulfate precipitation and dialyzed against PBS were used for preincubation of virions for FcR-dependent virion uptake (see Fig. 5) and virus neutralization (see Fig. 7). Rat anti-mouse CD68 MAb (clone FA-11), APC-conjugated rat anti-mouse F4/80 MAb (clone BM8), and APC-conjugated hamster anti-mouse CD11c MAb (clone N418) were from BioLegend. Rabbit anti-GFP polyclonal antibody (pAb) was from Abcam (catalog number ab6556), goat anti-mouse podoplanin pAb was from R&D Systems (catalog number AF3244), and rat anti-mouse CD16/32 MAb (clone 2.4G2) was from BD Biosciences. Alexa Fluor 488 goat anti-rabbit IgG(H+L), Alexa Fluor 568 donkey anti-goat IgG(H+L), Alexa Fluor 633 goat anti-mouse IgG(H+L), and Alexa Fluor 633 goat anti-rat IgG(H+L) were from Invitrogen. Horseradish peroxidase-conjugated rabbit anti-mouse Ig pAb was from DakoCytomation, and alkaline phosphatase-conjugated goat anti-mouse IgG pAb was from Southern Biotech.

### Structure prediction of MuHV-4 gB.

Three-dimensional (3D) structure prediction of the MuHV-4 gB ectodomain (amino acids 32 to 730) was made using the iterative threading assembly refinement (I-TASSER) server (37, 38). Structure models were analyzed using PyMol (DeLano Scientific LLC).

### Western blot analysis and deglycosylation of virion gB.

Equal volumes of each virus stock (1 μl, corresponding to approximately 10^5^ PFU) were lysed in 20 μl protein loading buffer (PLB) (39) and boiled for 10 min. SDS-PAGE and Western blotting were done as described previously (36). Enzymatic deglycosylation was done using the GlycoPro enzymatic deglycosylation kit for N-linked and simple O-linked glycans (ProZyme) and recombinant endoglycosidase H (EndoH; New England BioLabs) according to the manufacturer's instructions. Briefly, virions (1 μl, corresponding to approximately 10^5^ PFU) were denatured in denaturation buffer for 10 min at 100°C, followed by deglycosylation with the corresponding enzymes for 3 h at 37°C. After addition of equal volumes of 2× PLB, the samples were processed for SDS-PAGE and Western blotting.

### Staining of infected cells for flow cytometry.

BHK-21 cells were infected at an MOI of 2 PFU/cell for 18 h. After trypsinization and one wash in PBS, the cells were stained with hybridoma supernatants diluted 1:2 in PBS containing 3% bovine serum albumin (BSA) for 1 h at 4°C. After one wash in PBS, the cells were incubated with Alexa Fluor 633 goat anti-mouse IgG (Invitrogen) diluted 1:2,000 in PBS containing 5% normal goat serum for 1 h at 4°C. After one wash in PBS, the cells were analyzed on a FACSCanto flow cytometer (BD Biosciences).

### ELISA on infected cells.

NMuMG cells were allowed to adhere overnight to 96-well plates before precooling for 1 h at 4°C, followed by incubation with MuHV-4 virions diluted in ice-cold complete medium for 2 h at 4°C. The cells were then washed 3 times in ice-cold PBS to remove unbound virions and either fixed directly or first incubated at 37°C in complete medium to allow virion endocytosis. After one wash in ice-cold PBS, cells were fixed by addition of ice-cold 4% formaldehyde in PBS and being left at room temperature (RT) for 1 h. Fixation was then stopped by incubation with 0.1 M glycine in PBS (15 min, RT), followed by 3 washes in PBS. The cells were then permeabilized with 0.1% Triton X-100 in PBS (30 min, RT), blocked with 2% BSA-0.1% Tween 20 in PBS (overnight, 4°C), and then incubated with primary MAbs (hybridoma supernatants) diluted 1:2 in 2% BSA-0.1% Tween 20 in PBS (3 h, RT), followed by 3 washes in PBS-0.1% Tween 20. The plates were then incubated with alkaline phosphatase-conjugated goat anti-mouse IgG pAb (Southern Biotech) diluted 1:1,000 in 2% BSA-0.1% Tween 20 in PBS (3 h, RT), followed by 6 washes in PBS-0.1% Tween 20. Bound secondary antibodies were detected by incubation with Sigma Fast *p*-nitrophenyl phosphate substrate (Sigma-Aldrich) and reading the absorbance at a 405-nm wavelength on a Sunrise microplate reader (Tecan). All absorbance values were normalized to the values measured for virus bound at 4°C.

### *In vivo* luciferase imaging.

Luciferase imaging of infected mice was essentially performed as described previously (20). Briefly, 1.5 mg/mouse d-luciferin (Caliper Life Sciences) diluted in 100 μl PBS was applied by intraperitoneal injection 5 min before imaging in an IVIS Lumina imager (Caliper Life Sciences) under isoflurane anesthesia. Mice were imaged from the ventral, dorsal, and left lateral sides for 1 min each. Data are shown as the total flux of photons per second in the area of the lung (sum of dorsal and ventral signals), the superficial cervical lymph nodes (SCLN) (ventral signal), or the spleen (left lateral signal).

### Titration of virus in infected lungs.

The lungs of infected mice were harvested and stored in complete medium at −70°C until analysis. The thawed tissues were then homogenized in complete medium with a blender, added to BHK-21 monolayers, and incubated for 2 h at 37°C before being overlaid with complete medium containing 0.3% carboxymethyl cellulose. After 4 days of culture, the cells were washed once in PBS, fixed with 4% formaldehyde in PBS, and stained with 0.1% toluidine blue.

### Infectious center assay.

Spleens from infected mice were homogenized in Griffiths tubes, filtered through a 70-μm strainer, added to BHK-21 monolayers, and incubated for 2 h at 37°C before being overlaid with complete medium containing 0.3% carboxymethyl cellulose. After 4 days of culture, the cells were washed once in PBS, fixed with 4% formaldehyde in PBS, and stained with 0.1% toluidine blue.

### qPCR for MuHV-4 DNA.

DNA was extracted from lymph node or spleen suspensions with the Wizard genomic DNA purification kit (Promega). Eighty nanograms of cellular DNA was then analyzed by quantitative PCR (qPCR) on a Rotor-Gene 3000 thermal cycler (Corbett Research) using HotStarTaq DNA polymerase (Qiagen). To quantify MuHV-4 DNA, part of the M2 open reading frame (ORF) (genomic coordinates 4166 to 4252) was amplified with primers M2 S (GTCAGTCGAGCCAGAGTCCAAC) and M2 R (ATCTATGAAACTGCTAACAGTGAACC). The PCR product was quantified by hybridization with probe M2 TM (TCCAGCCAATCTCTACGAGGTCCTTAATGA) labeled with 6-carboxyfluorescein (FAM) (5′) and 6-carboxytetramethylrhodamine (TAMRA) (3′). Amplification of serial dilutions of the MuHV-4 HindIII E fragment (genomic coordinates 107 to 6263) cloned into plasmid pBR322 (40) served as a standard curve. To quantify the cellular DNA, part of the adenosine phosphoribosyl transferase (APRT) gene was amplified with primers APRT F (GGGGCAAAACCAAAAAAGGA) and APRT A (TGTGTGTGGGGCCTGAGTC) and quantified with probe APRT TM (TGCCTAAACACAAGCATCCCTACCTCAA) labeled with Cy5 (5′) and BlackBerry quencher (BBQ) (3′). Amplification of serial dilutions of the PCR product cloned into plasmid pGEM-T Easy (Promega) served as a standard curve. The MuHV-4 genome copy numbers were then normalized to the APRT copy numbers.

### Immunohistofluorescence.

Lungs were fixed in PBS containing 1% formaldehyde, 10 mM sodium periodate, and 75 mM l-lysine (24 h, 4°C); equilibrated in 30% sucrose (18 h, 4°C); and then frozen in OCT and sectioned (7 μm) on a cryostat. Sections were air dried (2 h, RT) and blocked with PBS containing 2% BSA and 2% normal goat serum (1 h, RT). Sections were then incubated with primary antibodies diluted in PBS containing 2% BSA and 2% normal goat serum for 18 h at RT. Virus-expressed eGFP was detected with a rabbit pAb (Abcam), CD68 was detected with a rat MAb (clone FA-11; BioLegend), and podoplanin was detected with a goat pAb (R&D Systems). After incubation, sections were washed 3 times in PBS and then incubated (1 h, RT) with Alexa Fluor 568 donkey anti-goat IgG (Invitrogen). After 3 washes in PBS, potential free binding sites on the donkey anti-goat IgG conjugate were blocked by incubation (30 min, RT) with 2% normal goat serum. After 3 washes in PBS, the sections were incubated (1 h, RT) with a combination of Alexa Fluor 488 goat anti-rabbit IgG and Alexa Fluor 633 goat anti-rat IgG (Invitrogen). After 3 further washes in PBS, the sections were mounted in Prolong Gold containing 4′,6-diamidino-2-phenylindole (DAPI; Invitrogen). Fluorescence was visualized using a Leica SP5 confocal laser scanning microscope, and images were analyzed with ImageJ.

### Statistical analysis.

Unless indicated otherwise, the pooled values of the gB FCS^−^ viruses (ΔFCSv1, ΔFCSv2, and ΔFCSv3) were compared to the pooled values of the gB FCS^+^ viruses (WT, ΔFCSv1 R, ΔFCSv2 R, and ΔFCSv3 R) by Student's *t* test on Microsoft Excel 2010 using the following settings: two-tailed distribution and two-sample unequal variance. *P* values of <0.05 were considered statistically significant and are mentioned in the figure legends.

## RESULTS

### Mutation of the furin cleavage site of MuHV-4 gB.

To investigate the role of gB cleavage in MuHV-4 infection, we mutated its canonical FCS, ^431^R-R-K-R^434^, located in a short loop within domain II (Fig. 1A). Three different mutations were introduced: first, deletion of the entire motif (ΔFCSv1); second, replacement by a ^431^G-T-G-G^434^ motif (ΔFCSv2); and third, replacement by a ^431^G-T^432^ motif (ΔFCSv3) (Fig. 1A). The mutations were introduced into the background of a recombinant MuHV-4 strain 68 expressing firefly luciferase from an early lytic cycle MuHV-4 promoter (M3-LUC), which allows *in vivo* imaging of infected mice without detectable virus attenuation (20). Mutagenesis of the bacterial artificial chromosome (BAC)-cloned M3-LUC MuHV-4 genome was performed by RecA-mediated homologous recombination in E. coli as previously described (29, 30). The recombinant BACs containing the ΔFCSv2 and ΔFCSv3 mutations, including the diagnostic KpnI site, were identified by KpnI digestion and agarose gel electrophoresis (not shown). Since the ΔFCSv1 mutation does not include a diagnostic restriction site, we identified the ΔFCSv1 mutant BACs with a diagnostic PCR spanning the FCS (primers P1 and P2 [Fig. 1B]) (not shown). The overall integrity of the mutant and revertant BACs was verified by digestion with HindIII or EcoRI and agarose gel electrophoresis (Fig. 1C and data not shown). After reconstitution of virus by transfection of the BACs into BHK-21 cells, we again verified that the mutant viruses carried the desired mutations. To this end, we analyzed viral DNA by KpnI digestion and Southern blot analysis to identify the ΔFCSv2 and ΔFCSv3 mutations and by the diagnostic PCR spanning the FCS to identify the ΔFCSv1 mutation. Southern blot analysis confirmed that the ΔFCSv2 and ΔFCSv3 mutant viruses showed two KpnI fragments, 3.2 and 2.4 kb, as opposed to the wild-type (WT), ΔFCSv1, and revertant viruses, which showed a single 5.6-kb KpnI fragment (Fig. 1D). The diagnostic PCR confirmed that the ΔFCSv1 and the ΔFCSv3 mutants showed shorter PCR products than did the WT, ΔFCSv2, and revertant viruses, consistent with the deletion of 12 bp in the ΔFCSv1 mutant and 6 bp in the ΔFCSv3 mutant (Fig. 1E).

### Deletion of the furin cleavage site abolishes cleavage of gB.

**Fig 2:**
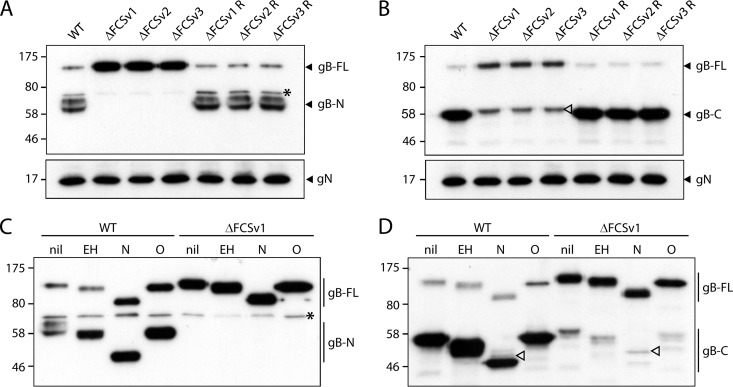
Western blot analysis of mutant virion gB. (A) Virions of eGFP^+^ WT virus and ΔFCSv1, ΔFCSv2, and ΔFCSv3 mutant and revertant viruses were analyzed by Western blotting with the gB-specific MAb MG-2C10 recognizing an epitope N terminal of the FCS. Detection of glycoprotein N (gN) with MAb 3F7 served as a loading control. All three mutant viruses, ΔFCSv1, ΔFCSv2, and ΔFCSv3, showed only full-length gB (gB-FL, 120 kDa) but no N-terminal cleavage product (gB-N, 65 kDa). The asterisk denotes an unspecific band. Equivalent data were obtained in a repeat experiment. The positions of size standards (in kDa) are shown to the left of the blots. (B) Virions were analyzed as in panel A but probed with the gB-specific MAb MG-4D11 recognizing an epitope C terminal of the FCS. The mutant viruses showed only gB-FL but no C-terminal furin cleavage product (gB-C, 55 kDa). The weak 60-kDa bands observed for the mutant viruses (empty arrowhead) differ in size from the 55-kDa C-terminal furin cleavage products and appear to be hidden by the latter in the WT and revertant viruses. Equivalent data were obtained in a repeat experiment. (C) Virions of eGFP^+^ WT and ΔFCSv1 mutant viruses were subjected to enzymatic deglycosylation with endoglycosidase H (EH), N-glycanase/PNGase F (N), or sialidase A and O-glycanase (O) and analyzed by Western blotting with MAb MG-2C10 recognizing gB-N. The asterisk denotes an unspecific band. Equivalent data were obtained for the ΔFCSv2 mutant and the ΔFCSv2 revertant viruses. (D) Virions were treated and analyzed as for panel C, except that gB was detected with MAb MG-4D11 recognizing gB-C. Upon removal of N-linked glycans, the size difference between the weak 60-kDa band observed for the mutant viruses (open arrowhead) and the 55-kDa gB-C band becomes more apparent. Equivalent data were obtained for the ΔFCSv2 mutant and the ΔFCSv2 revertant viruses.

The effect of the FCS mutations on gB cleavage was assessed by Western blotting for virion gB. A MAb (MG-2C10) recognizing an epitope N terminal of the FCS detected in WT and revertant viruses a weak 120-kDa band—full-length gB—and a strong 65-kDa band—its N-terminal gB cleavage product (Fig. 2A). In contrast, only full-length gB was detected in the mutant viruses (Fig. 2A), demonstrating that the FCS mutations completely abolished gB cleavage. The 65-kDa band appears as a doublet because it comigrates with the bovine serum albumin (66 kDa) present in virion preparations. A MAb (MG-4D11) recognizing an epitope C terminal of the FCS similarly recognized mainly cleaved gB (55 kDa) with WT and revertant viruses and only the full-length form in the FCS mutants (Fig. 2B).

The weak 60-kDa band preserved in the mutant viruses (open arrowhead in Fig. 2B) appeared not to be gB because it differs in size from the 55-kDa C-terminal cleavage product. We confirmed this for the ΔFCSv1 mutant by enzymatic deglycosylation prior to Western blotting (Fig. 2C and D). Full-length gB showed similar endoglycosidase H (EndoH) sensitivity and similar extents of N-linked (N-glycanase/peptide-*N*-glycosidase F [PNGase F] treatment) and O-linked (sialidase A plus O-glycanase treatment) glycosylation between WT and ΔFCSv1 mutant viruses. Enzymatic removal of N-linked glycans made clear that the 60-kDa band observed for the mutant viruses in Fig. 2B (open arrowhead) was also present in the WT virus but hidden by the strong gB-specific 55-kDa band (Fig. 2D, open arrowheads). We presume that this band reflected weak cross-reactivity of MAb MG-4D11 with an unrelated glycoprotein.

### Uncleaved gB shows no obvious change in antigenicity.

**Fig 3:**
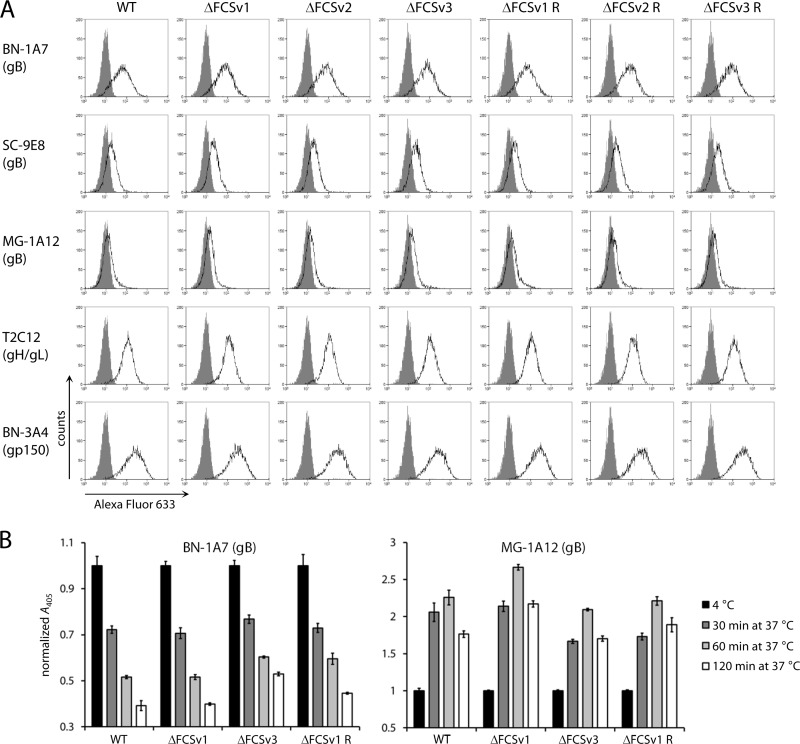
Antigenic conformation of uncleaved gB. (A) BHK-21 cells were infected with eGFP^+^ WT virus and ΔFCSv1, ΔFCSv2, and ΔFCSv3 mutant and revertant viruses at an MOI of 2 PFU/cell. Eighteen hours later, cells were stained with MAbs BN-1A7 and SC-9E8 recognizing prefusion gB, MAb MG-1A12 recognizing postfusion gB, MAb T2C12 recognizing gH/gL, and MAb BN-3A4 recognizing gp150. Bound antibody was detected with an Alexa Fluor 633-conjugated secondary antibody and flow cytometry (open histograms). The gray histograms show staining with secondary antibody only. Detection of gH/gL and gp150 served as the control for equivalent infection of cells. Equivalent data were obtained in a repeat experiment. (B) NMuMG cells were incubated with eGFP^+^ WT, ΔFCSv1 and ΔFCSv3 mutant, and ΔFCSv1 revertant viruses at an MOI of 15 eGFP units/cell (2 h, 4°C). After 3 washes in ice-cold PBS to remove unbound virions, the cells were fixed either directly or after further incubations at 37°C to allow virion endocytosis. The cells were then incubated with MAb BN-1A7 recognizing prefusion gB and MAb MG-1A12 recognizing postfusion gB. Bound antibody was detected with an alkaline phosphatase-conjugated secondary antibody, incubation with *p*-nitrophenyl phosphate substrate, and measurement of the absorbance at 405 nm (*A*_405_). The bars show means ± standard errors of the means from 6 wells. Equivalent data were obtained in 3 further experiments.

gB is antigenically different between extracellular virions (i.e., prefusion) and after capsid and tegument release (i.e., postfusion). Prefusion gB is recognized by MAbs BN-1A7 and SC-9E8 and not by MAb MG-1A12; postfusion gB is recognized by MAb MG-1A12 and not by MAbs BN-1A7 and SC-9E8 (26, 33). To determine whether FCS mutation affected the stability of prefusion gB, we stained the surface of infected BHK-21 cells with MAbs BN-1A7, SC-9E8, and MG-1A12, followed by flow cytometry. Staining with MAbs T2C12 recognizing gH/gL and BN-3A4 recognizing gp150 controlled for equivalent infection of cells. As shown in Fig. 3A, the ΔFCSv1, ΔFCSv2, and ΔFCSv3 mutants displayed the same staining as did WT and the revertant viruses.

To determine if FCS mutation affected the antigenic transition of prefusion into postfusion gB during cell entry, we performed a kinetic analysis of virion entry by ELISA on cell monolayers (36). A sensitive assessment of the changes in virion antigenicity during cell entry requires a synchronous entry of the whole virion population. These experiments were conducted in NMuMG epithelial cells, because virion entry is most efficient and synchronous in this cell line. To this end, virions were bound to cells at 4°C, unbound virions were removed by washing, and the cells were fixed either directly or after further incubations at 37°C to allow virion endocytosis. The gB on virions bound to cells at 4°C is in the prefusion conformation (BN-1A7^+^ MG-1A12^−^), while after incubation for 2 h at 37°C, capsid and tegument have been released and gB is in the postfusion conformation (BN-1A7^−^ MG-1A12^+^) (33). As shown in Fig. 3B, the ΔFCSv1 and ΔFCSv3 mutant viruses displayed similar kinetics of BN-1A7 epitope loss and MG-1A12 epitope gain as did the WT and ΔFCSv1 revertant viruses. Together, these data demonstrate that FCS mutation does not affect the stability of prefusion gB and its transition into postfusion gB during cell entry.

### Growth properties of gB FCS^−^ viruses.

**Fig 4:**
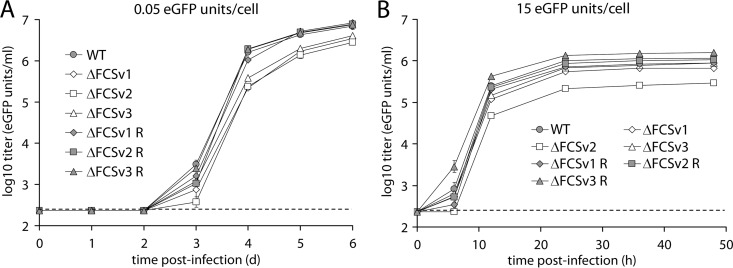
Growth properties of gB FCS^−^ viruses. (A) Low-MOI growth curve. BHK-21 cells were infected with eGFP^+^ WT virus and ΔFCSv1, ΔFCSv2, and ΔFCSv3 mutant and revertant viruses at an MOI of 0.05 eGFP units/cell. At the indicated times p.i., the cells were lysed by 1 cycle of freeze-thawing and titrated on BHK-21 cells by counting eGFP^+^ cells by flow cytometry. Each point shows the mean ± standard error of the mean from 3 wells. The dashed line shows the sensitivity threshold of virus titration. The titers of the gB FCS^−^ viruses (ΔFCSv1, ΔFCSv2, and ΔFCSv3) were significantly lower than those of the gB FCS^+^ viruses (WT, ΔFCSv1 R, ΔFCSv2 R, and ΔFCSv3 R) on days 3 (*P* < 0.006 by Student's *t* test), 4 (*P* < 10^−10^), 5 (*P* < 10^−7^), and 6 (*P* < 10^−6^) p.i. Equivalent data were obtained in a repeat experiment. (B) High-MOI growth curve. BHK-21 cells were infected with eGFP^+^ WT virus and ΔFCSv1, ΔFCSv2, and ΔFCSv3 mutant and revertant viruses at an MOI of 15 eGFP units/cell. At the indicated times p.i., the cells were lysed by 1 cycle of freeze-thawing and titrated on BHK-21 cells by counting eGFP^+^ cells by flow cytometry. Each point shows the mean ± standard error of the mean from 3 wells. The dashed line shows the sensitivity threshold of virus titration. The titers of the ΔFCSv1 and ΔFCSv3 mutants were marginally but significantly reduced compared to the gB FCS^+^ viruses at 12 (*P* < 10^−5^ by Student's *t* test), 24 (*P* < 10^−4^), 36 (*P* < 10^−4^), and 48 (*P* < 0.001) h p.i. The titers of the ΔFCSv2 mutant were more substantially reduced compared to the gB FCS^+^ viruses at 6 (*P* < 0.001), 12 (*P* < 10^−10^), 24 (*P* < 10^−6^), 36 (*P* < 10^−10^), and 48 (*P* < 0.05) h p.i. The titers of the ΔFCSv2 mutant were also significantly lower than those of the ΔFCSv1 and ΔFCSv3 mutants at 6 (*P* < 0.002), 12 (*P* < 10^−6^), 24 (*P* < 10^−4^), and 36 (*P* < 10^−6^) h p.i. Equivalent data were obtained in a repeat experiment.

To address the effect of the gB FCS mutations on viral replication, we performed growth curves in BHK-21 fibroblasts. Upon low-MOI infection (0.05 enhanced green fluorescent protein [eGFP] units/cell), all gB FCS^−^ viruses (ΔFCSv1, ΔFCSv2, and ΔFCSv3) showed moderately but significantly reduced growth compared to the gB FCS^+^ viruses (WT, ΔFCSv1 R, ΔFCSv2 R, and ΔFCSv3 R) (Fig. 4A). Upon high-MOI infection (15 eGFP units/cell), the ΔFCSv1 and ΔFCSv3 mutants grew only marginally less well than the gB FCS^+^ viruses, while the ΔFCSv2 mutant showed a more substantial growth defect (Fig. 4B). Since the growth defect was significantly greater for the ΔFCSv2 than for the ΔFCSv1 and ΔFCSv3 mutants, we concluded that the observed phenotype of the ΔFCSv2 mutant is due not to lack of gB cleavage but perhaps to an unspecific effect of its introduced G-T-G-G sequence. The reason for this remains unclear, especially since ΔFCSv2 is the only mutation to retain the correct spacing within the gB molecule (Fig. 1A). This mutant was therefore excluded from further analysis. Together, these findings show that lack of gB cleavage moderately reduces multistep virus growth, while it only marginally affects single-step growth.

### gB FCS^−^ viruses show impaired myeloid cell infection.

**Fig 5:**
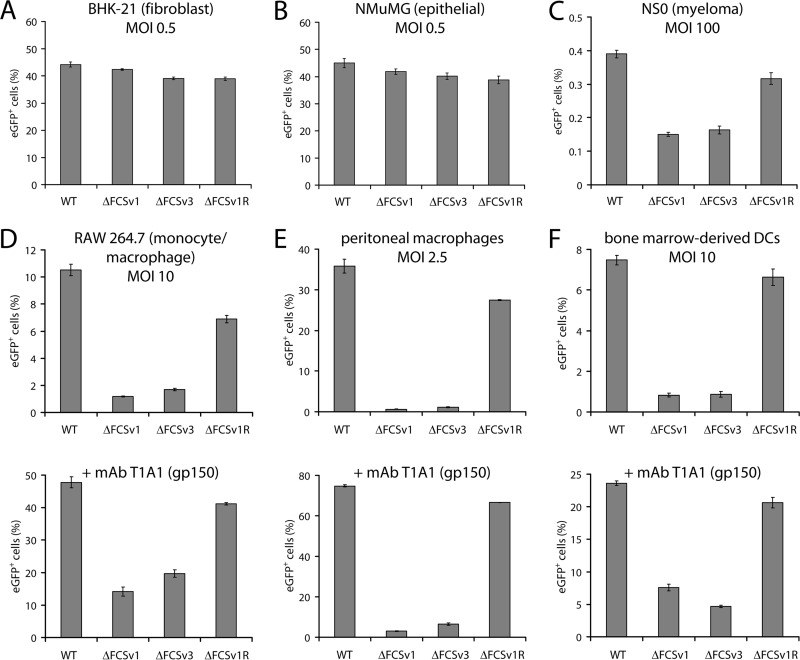
Comparative analysis of virus entry in fibroblasts, epithelial cells, myeloma cells, macrophages, and DCs. (A and B) BHK-21 fibroblasts (A) or NMuMG epithelial cells (B) were infected with eGFP^+^ WT, ΔFCSv1 and ΔFCSv3 mutant, and ΔFCSv1 revertant viruses at an MOI of 0.5 eGFP units/cell. After incubation in the presence of 100 μg/ml PAA for 18 h at 37°C, the proportion of eGFP^+^ cells was determined by flow cytometry. The bars show the means ± standard errors of the means from 3 wells. Equivalent data were obtained in 3 further experiments. (C) NS0 myeloma cells were infected as for panel A but at an MOI of 100 eGFP units/cell. The bars show the means ± standard errors of the means from 3 wells. The values for the gB FCS^−^ viruses (ΔFCSv1 and ΔFCSv3) were significantly lower than those for the gB FCS^+^ viruses (WT and ΔFCSv1 R) (*P* < 10^−4^ by Student's *t* test). Equivalent data were obtained in a repeat experiment. (D) RAW 264.7 monocytes/macrophages were infected with eGFP^+^ WT, ΔFCSv1 and ΔFCSv3 mutant, and ΔFCSv1 revertant viruses at an MOI of 10 eGFP units/cell in the absence or presence of the gp150-specific MAb T1A1 (100 μg/ml). After incubation in the presence of 100 μg/ml PAA for 18 h at 37°C, the cells were treated with 1 μg/ml lipopolysaccharide for 6 h to activate viral eGFP expression. The proportion of eGFP^+^ cells was then determined by flow cytometry. The bars show the means ± standard errors of the means from 3 wells. The values for the gB FCS^−^ viruses were significantly lower than those for the gB FCS^+^ viruses in the absence (*P* < 0.001 by Student's *t* test) and in the presence (*P* < 10^−6^) of MAb T1A1. Equivalent data were obtained in 3 further experiments. (E) Peritoneal cavity cells were infected as for panel D but at an MOI of 2.5 eGFP units/cell. After incubation in the presence of 100 μg/ml PAA for 18 h at 37°C, the cells were treated with 1 μg/ml lipopolysaccharide for 6 h to activate viral eGFP expression. The proportion of eGFP^+^ peritoneal macrophages (F4/80^high^) was then determined by flow cytometry. The bars show the means ± standard errors of the means from 3 wells. The values for the gB FCS^−^ viruses were significantly lower than those for the gB FCS^+^ viruses in the absence (*P* < 10^−4^ by Student's *t* test) and in the presence (*P* < 10^−8^) of MAb T1A1. Equivalent data were obtained in a repeat experiment. (F) Bone marrow-derived DCs were infected as for panel D. After incubation in the presence of 100 μg/ml PAA for 18 h at 37°C, the cells were treated with 250 ng/ml lipopolysaccharide for 6 h to activate viral eGFP expression. The proportion of eGFP^+^ DCs (CD11c^high^) was then determined by flow cytometry. The bars show the means ± standard errors of the means from 3 wells. The values for the gB FCS^−^ viruses were significantly lower than those for the gB FCS^+^ viruses in the absence (*P* < 10^−5^ by Student's *t* test) and in the presence (*P* < 10^−7^) of MAb T1A1. Equivalent data were obtained in a repeat experiment.

MuHV-4 host colonization involves at least four cell types: epithelial cells for lytic replication in the lung (21), DCs and macrophages for transport to lymph nodes and transmission to B cells (19, 41), and B cells for systemic dissemination and persistence (42, 43). We tested the capacity of gB FCS^−^ viruses to infect each of these cell types *in vitro*. Infection was assessed by eGFP expression from eGFP^+^ versions of WT, ΔFCSv1 and ΔFCSv3 mutant, and ΔFCSv1 revertant viruses. We included phosphonoacetic acid (PAA) in the cultures to prevent viral DNA replication and thus any secondary spread. After addition of viruses, the cells were incubated overnight and the proportion of eGFP^+^ cells was then determined by flow cytometry. Input virus titers were determined on BHK-21 fibroblasts; their infection therefore controlled for equivalent virus doses. The gB FCS^+^ and FCS^−^ virus stocks had similar titers (not shown) and protein contents (Fig. 2); infection at identical MOIs therefore involved the addition of similar virion amounts. Figure 5A and B show that NMuMG epithelial cell infection exactly mirrored BHK-21 cell infection. Thus, the mutant viruses had no obvious defect in epithelial infection. They did show a defect in the infection of the NS0 myeloma cell line (Fig. 5C). However, *in vitro* B cell infection by MuHV-4 is poorly efficient, yielding here only 0.4% eGFP^+^ cells at 100 fibroblast eGFP units/cell. Thus, although the significance of the gB FCS^−^ defect was hard to judge, it nonetheless indicates that cleavage of gB may have some role in B cell infection.

gB FCS^−^ viruses showed a marked infection defect for RAW 264.7 monocytes/macrophages (Fig. 5D). This was confirmed for *ex vivo* peritoneal macrophages, which are infected better than RAW 264.7 cells (Fig. 5E), and for bone marrow-derived DCs, which are infected somewhat less well (Fig. 5F). Thus, gB FCS^−^ MuHV-4 appears to have a general defect in myeloid infection.

The main barrier to myeloid cell infection by MuHV-4 is poor binding, and infection is markedly increased by opsonization with viral glycoprotein-specific antibodies that allow binding to endocytic myeloid immunoglobulin Fc receptors (FcRs) (44). To test if the gB FCS^−^ viruses show a similar deficit for entry by this route, we preincubated virions with a nonneutralizing MAb directed against gp150 (T1A1). This enhanced macrophage and DC infection, but a substantial, although somewhat less pronounced, gB FCS^−^ infection defect remained (Fig. 5D to F).

### Lack of gB cleavage does not affect cell binding and virion endocytosis.

**Fig 6:**
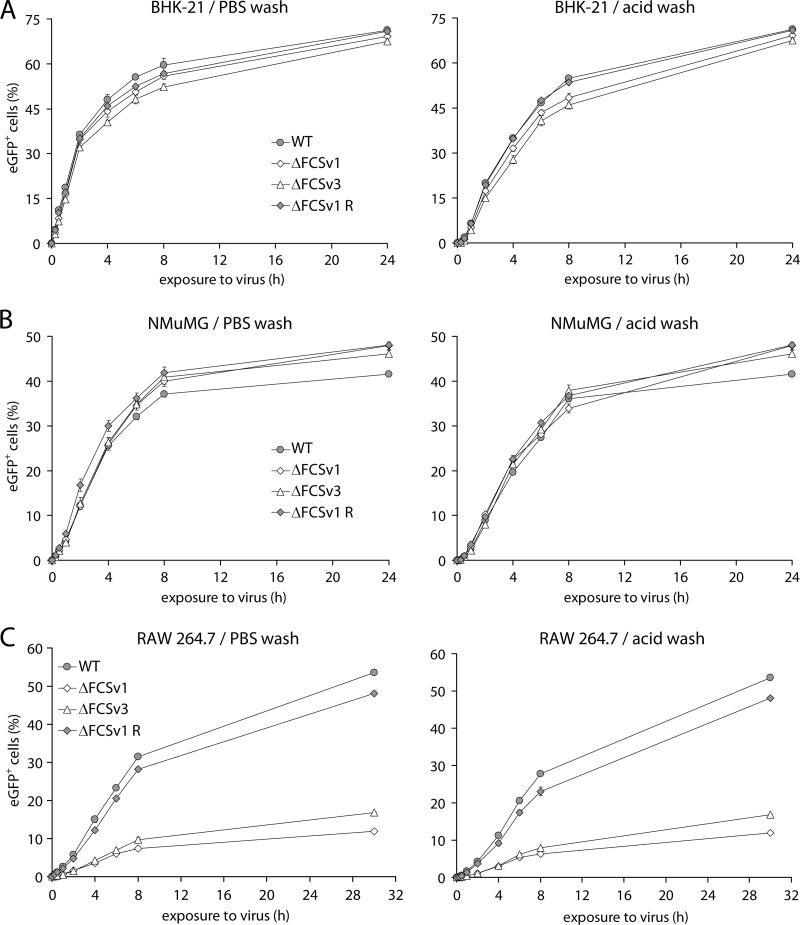
Kinetics of virus entry in fibroblasts, epithelial cells, and macrophages. (A) BHK-21 fibroblasts were exposed at 37°C to eGFP^+^ WT, ΔFCSv1 and ΔFCSv3 mutant, and ΔFCSv1 revertant viruses at an MOI of 1 eGFP units/cell in the presence of 100 μg/ml PAA for the times indicated on the *x* axis. The cells were then washed with PBS to remove unbound virions or with phosphate-citrate buffer (pH 3) (acid wash) to inactivate all extracellular virions, followed by incubation at 37°C in the presence of 100 μg/ml PAA until 24 h p.i. The proportion of eGFP^+^ cells was then determined by flow cytometry. Each point shows the mean ± standard error of the mean from 3 wells. The incubation times required for half-maximal infection levels (*t*_50%_) were as follows (mean ± standard error of the mean): PBS wash, 2 ± 0.05 h for gB FCS^+^ (WT and ΔFCSv1 R) and 2.3 ± 0.1 h for gB FCS^−^ (ΔFCSv1 and ΔFCSv3) viruses; acid wash, 4.1 ± 0.08 h for gB FCS^+^ and 4.7 ± 0.15 h for gB FCS^−^ viruses. For acid wash, the difference in *t*_50%_ values was statistically significant (*P* < 0.01 by Student's *t* test). Equivalent data were obtained in a repeat experiment. (B) NMuMG epithelial cells were infected and analyzed as for panel A. The *t*_50%_ values were as follows (mean ± standard error of the mean): PBS wash, 3.2 ± 0.09 h for gB FCS^+^ and 3.6 ± 0.11 h for gB FCS^−^ viruses; acid wash, 4.3 ± 0.08 h for gB FCS^+^ and 4.5 ± 0.14 h for gB FCS^−^ viruses. For PBS wash, the difference in *t*_50%_ values was statistically significant (*P* < 0.02 by Student's *t* test). Equivalent data were obtained in a repeat experiment. (C) RAW 264.7 monocytes/macrophages were exposed at 37°C to eGFP^+^ WT, ΔFCSv1 and ΔFCSv3 mutant, and ΔFCSv1 revertant viruses at an MOI of 20 eGFP units/cell in the presence of 100 μg/ml PAA for the times indicated on the *x* axis. The cells were then washed with PBS or phosphate-citrate buffer (pH 3) (acid wash), followed by incubation at 37°C in the presence of 100 μg/ml PAA. At 24 h p.i., the cells were treated with 1 μg/ml lipopolysaccharide for 6 h to activate viral eGFP expression. The proportion of eGFP^+^ cells was then determined by flow cytometry. Each point shows the mean ± standard error of the mean from 3 wells. The *t*_50%_ values were as follows (mean ± standard error of the mean): PBS wash, 6.9 ± 0.02 h for gB FCS^+^ and 6.5 ± 0.28 h for gB FCS^−^ viruses; acid wash, 8.3 ± 0.47 h for gB FCS^+^ and 8.3 ± 0.5 h for gB FCS^−^ viruses. Equivalent data were obtained in a repeat experiment.

In Fig. 5, overnight incubation with virus allowed ample time for binding and endocytosis. To investigate more specifically whether a lack of gB cleavage affected these processes, BHK-21 fibroblasts (Fig. 6A), NMuMG epithelial cells (Fig. 6B), and RAW 264.7 monocytes/macrophages (Fig. 6C) were exposed for increasing times to eGFP^+^ WT, ΔFCSv1 and ΔFCSv3 mutant, or ΔFCSv1 revertant viruses before being washed with PBS to remove unbound virions or with phosphate-citrate buffer (pH 3) (acid wash) to inactivate all extracellular virions. After washing, all the cells were cultured overnight to allow eGFP expression and analyzed by flow cytometry.

In BHK-21 (Fig. 6A) and NMuMG cells (Fig. 6B), the entry kinetics looked similar for the ΔFCSv1 and ΔFCSv3 mutants and the WT and ΔFCSv1 revertant viruses. However, when the exposure times required for half-maximal infection levels (*t*_50%_) were compared between the gB FCS^−^ (ΔFCSv1 and ΔFCSv3) and gB FCS^+^ (WT and ΔFCSv1 R) viruses, the mean values were consistently moderately higher for the gB FCS^−^ than for the gB FCS^+^ viruses. However, in BHK-21 cells the difference was statistically significant only after acid wash, and in NMuMG cells, it was significant only after PBS wash. We therefore concluded that the lack of gB cleavage, if anything, only marginally affects virion binding and endocytosis in BHK-21 and NMuMG cells.

The same appeared to be true also for RAW 264.7 monocyte/macrophage infections: the relative deficits of the gB FCS^−^ viruses compared to the gB FCS^+^ viruses were similar irrespective of how long the cells were exposed to virus before the PBS or acid wash (Fig. 6C). This was confirmed by the observation that the *t*_50%_ values were not significantly different between the gB FCS^−^ and FCS^+^ viruses both after PBS wash and after acid wash. Thus, PBS or acid wash seems not to accentuate the deficit of the gB FCS^−^ viruses, suggesting that they bind normally to cells and are normally endocytosed from the cell surface. Their myeloid infection defect therefore seems to occur at a postendocytic entry step. As PRV and EBV gB cleavages are important for cell-to-cell fusion (11, 15), it seemed most likely that mutant MuHV-4 lacking gB cleavage was impaired in viral membrane fusion and thus in escape from the endosomes. We have previously studied these processes extensively in epithelial cells by immunofluorescence (26, 36, 45). However, the relative inefficiency of myeloid cell infection prevented equivalent analysis giving clear answers (data not shown). Thus, we could conclude only that gB FCS mutation impaired infection after virion binding and endocytosis.

### gB FCS disruption alters the virion neutralization profile.

**Fig 7:**
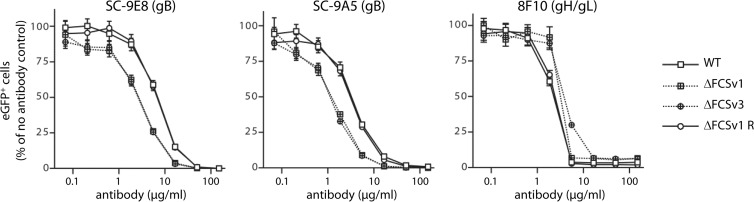
Susceptibility of gB FCS^−^ viruses to gB-directed neutralization. eGFP^+^ WT, ΔFCSv1 and ΔFCSv3 mutant, and ΔFCSv1 revertant viruses (MOI of 0.5 eGFP units/cell) were incubated (2 h, RT) with the gB-specific neutralizing MAbs SC-9E8 and SC-9A5 blocking viral membrane fusion or the gH/gL-specific MAb 8F10 blocking cell binding. The viruses were then added to BHK-21 cells and incubated for 18 h at 37°C in the presence of 100 μg/ml PAA. The eGFP^+^ cells were enumerated by flow cytometry and are shown relative to untreated virus. Each point shows the mean ± standard error of the mean from 3 wells. The gB FCS^−^ viruses (ΔFCSv1 and ΔFCSv3) were neutralized significantly better than the gB FCS^+^ viruses (WT and ΔFCSv1 R) with MAb SC-9E8 at antibody doses of 16.7 (*P* < 10^−6^ by Student's *t* test), 5.56 (*P* < 10^−6^), 1.85 (*P* < 10^−4^), 0.62 (*P* < 0.006), and 0.21 (*P* < 0.009) μg/ml. Similarly, the gB FCS^−^ viruses were neutralized significantly better than the gB FCS^+^ viruses with MAb SC-9A5 at antibody doses of 16.7 (*P* < 0.001), 5.56 (*P* < 10^−5^), 1.85 (*P* < 10^−6^), 0.62 (*P* < 0.001), and 0.21 (*P* < 0.02) μg/ml. In contrast, the gB FCS^−^ viruses were neutralized significantly less well than the gB FCS^+^ viruses by MAb 8F10 at antibody doses of 5.56 (*P* < 0.04) and 1.85 (*P* < 10^−4^) μg/ml. Equivalent data were obtained in 2 further experiments.

The MuHV-4 gB associates with gH/gL (46), and a loss of gL increases virion susceptibility to gB-directed neutralization (47). To test whether gB FCS disruption might also affect virion neutralization, we incubated eGFP^+^ WT, ΔFCSv1 and ΔFCSv3 mutant, and ΔFCSv1 revertant viruses with increasing concentrations of the gB-specific neutralizing MAbs SC-9E8 and SC-9A5 (26), then added them to BHK-21 cells, and, after overnight incubation in the presence of PAA, enumerated eGFP^+^ cells by flow cytometry. Incubation with the gH/gL-specific neutralizing MAb 8F10 was included as a control. Figure 7 shows that the ΔFCSv1 and ΔFCSv3 mutant viruses were significantly more susceptible to neutralization by MAbs SC-9E8 and SC-9A5 than were WT and ΔFCSv1 revertant viruses and significantly less susceptible to neutralization by MAb 8F10. This altered neutralization profile suggested a subtle change in the fusion complex, consistent with the idea that virion membrane fusion was impaired.

### gB FCS^−^ viruses show reduced lytic virus spread in the lungs.

**Fig 8:**
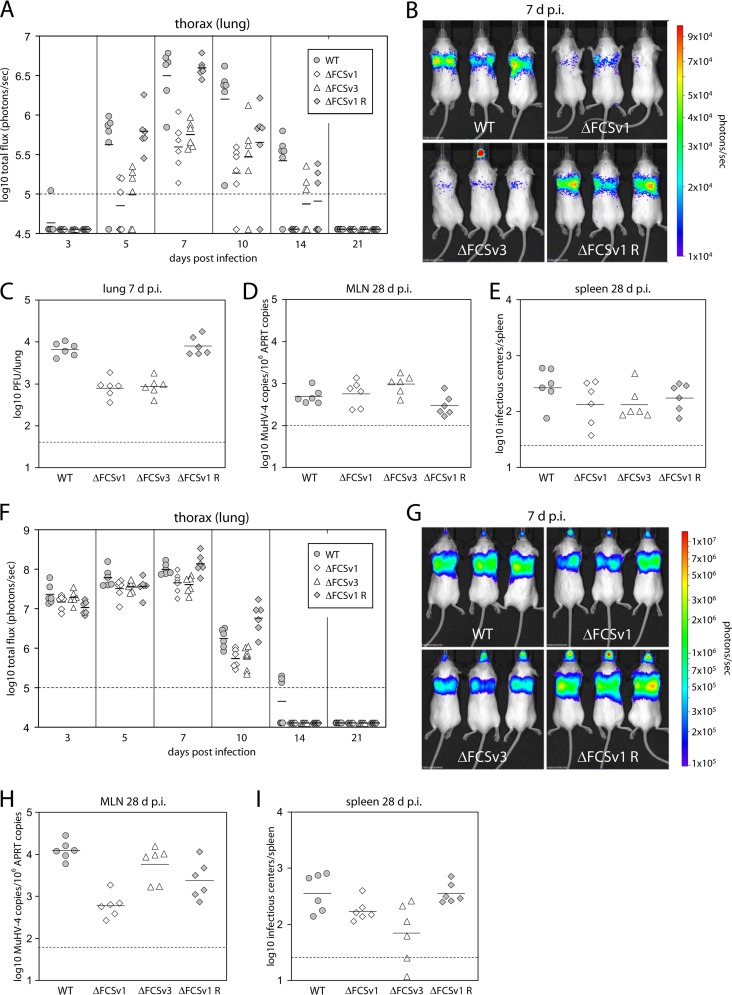
*In vivo* replication of gB FCS^−^ viruses. (A) Low-dose infection. BALB/c mice were infected intranasally with 10 PFU/mouse of BAC^−^ WT, ΔFCSv1 and ΔFCSv3 mutant, and ΔFCSv1 revertant viruses in a volume of 30 μl under general anesthesia. At the indicated time postinfection, viral replication was monitored by *in vivo* luciferase imaging. The total flux of the luciferase signals originating from the thorax is shown in photons/s. The symbols represent single mice, while the horizontal bars show the mean value from each group. (B) Representative images of infected mice at day 7 p.i. The signals for the gB FCS^−^ viruses (ΔFCSv1 and ΔFCSv3) were significantly lower than those for the gB FCS^+^ viruses (WT and ΔFCSv1 R) on days 5 (*P* < 10^−4^ by Student's *t* test), 7 (*P* < 0.10^−7^), 10 (*P* < 0.02), and 14 (*P* < 0.02) p.i. (C) Mice were infected as for panel A, but titers of recoverable virus in the lungs were measured by plaque assay on day 7 p.i. The titers for the gB FCS^−^ viruses were significantly lower than those for the gB FCS^+^ viruses (*P* < 10^−9^ by Student's *t* test). (D) Virus loads in the mediastinal lymph nodes (MLN) of the mice shown in panel A were measured by quantitative PCR on day 28 p.i. The values for the gB FCS^−^ viruses were subtly, but significantly, higher than those for the gB FCS^+^ viruses (*P* < 0.02 by Student's *t* test). (E) Levels of latent virus in the spleens of the mice shown in panel A were measured by infectious center assay on day 28 p.i. (F) High-dose infection. BALB/c mice were infected as for panel A but with a dose of 5,000 PFU/mouse. (G) Representative images of infected mice at day 7 p.i. The signals for the gB FCS^−^ viruses were significantly lower than those for the gB FCS^+^ viruses on days 7 (*P* < 10^−4^ by Student's *t* test) and 10 (*P* < 10^−4^) p.i. (H) Virus loads in the MLN of the mice shown in panel F were measured by quantitative PCR on day 28 p.i. (I) Levels of latent virus in the spleens of the mice shown in panel F were measured by infectious center assay on day 28 p.i. The values for the gB FCS^−^ viruses were subtly, but significantly, lower than those for the gB FCS^+^ viruses (*P* < 0.003 by Student's *t* test). In all panels, the dashed lines show the sensitivity thresholds of the respective assays.

Having established the effects of gB FCS mutation *in vitro*, we next assessed its consequences for host colonization. To this end, BALB/c mice were infected with BAC^−^ WT, ΔFCSv1 and ΔFCSv3 mutant, and ΔFCSv1 revertant viruses by intranasal inoculation. The viruses were constructed with a firefly luciferase reporter, so infection could be tracked by live imaging (20). After low-dose infection (10 PFU/mouse), luciferase signals from the thorax at days 5, 7, 10, and 14 postinfection (p.i.) were significantly lower for the gB FCS^−^ (ΔFCSv1 and ΔFCSv3) than for the gB FCS^+^ (WT and ΔFCSv1 R) viruses (Fig. 8A and B). Plaque assays at day 7 p.i. confirmed a significant replication defect of the gB FCS^−^ viruses in lungs (Fig. 8C). While the virus loads in mediastinal lymph nodes (MLN) at day 28 p.i. were marginally higher for the gB FCS^−^ than for the gB FCS^+^ viruses (Fig. 8D), the latency levels in spleens were not significantly different between gB FCS^−^ and gB FCS^+^ viruses (Fig. 8E). After high-dose infection (5,000 PFU/mouse), the gB FCS^−^ viruses showed a moderate, but again significant, lytic replication defect in lungs at days 7 and 10 p.i. (Fig. 8F and G). While the virus loads in MLNs on day 28 p.i. were not significantly different between gB FCS^−^ and gB FCS^+^ viruses (Fig. 8H), the latency levels in spleens were subtly but significantly lower for the gB FCS^−^ than for the gB FCS^+^ viruses (Fig. 8I). However, because the gB FCS^−^ viruses did not show any latency deficit upon low-dose infection (Fig. 8E), we concluded that they were not obviously impaired in establishment of normal long-term latency levels. In contrast, a lack of gB cleavage resulted in significantly reduced lytic replication in the lower respiratory tract.

### gB cleavage is important for infection of alveolar macrophages.

**Fig 9:**
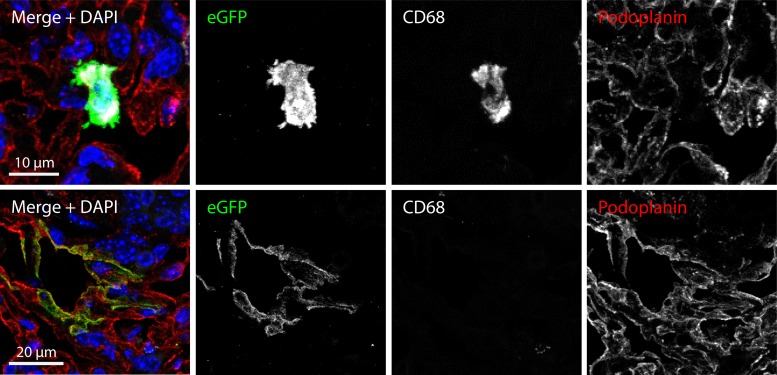
Histological analysis of infected lungs on day 1 p.i. BALB/c mice were infected intranasally with 10^6^ PFU/mouse of eGFP^+^ WT virus in a volume of 30 μl under general anesthesia. Lungs were taken 1 day later and processed for frozen sections. The sections were stained with antibodies specific for eGFP (green), CD68 (white), or podoplanin (red) and with DAPI (blue). Viral eGFP expression was detected in two cell populations. The major population consisted of individual round mononuclear cells which were CD68^+^, as well as F4/80^+^ and CD11c^+^ (not shown), consistent with them being alveolar macrophages. The minor population consisted of clusters of elongated cells lining the alveoli which stained positive for podoplanin, consistent with them being alveolar type I epithelial cells.

To understand better the *in vivo* infection defect, we analyzed lungs by immunostaining 1 day after virus inoculation (Fig. 9). We hypothesized that while later time points might show quantitative defects, reasons for such defects might be more apparent early in infection. To detect both lytic and latent infection, we stained for virus-expressed eGFP. MuHV-4 antigens have previously been identified in alveolar epithelial cells and large mononuclear cells 5 days after intranasal inoculation (21). We similarly detected virus-expressed eGFP in 2 main cell populations: single, round mononuclear cells and much larger, elongated cells—or clusters of cells—that lined the alveoli. The mononuclear cells were CD68^+^, F4/80^+^, and CD11c^+^ (Fig. 9 and data not shown), consistent with them being alveolar macrophages (48, 49), while the cells lining the alveoli stained positive for podoplanin (Fig. 9), consistent with them being alveolar type I epithelial cells (50). The numbers of infected alveolar epithelial cells were not significantly different between gB FCS^+^ (WT and ΔFCSv1 revertant) and gB FCS^−^ (ΔFCSv1 and ΔFCSv3) viruses, but gB FCS^−^ viruses infected significantly fewer alveolar macrophages (Table 1). Thus, gB cleavage was important for myeloid cell infection both *in vitro* and *in vivo*.

**Table 1:** Quantification of eGFP^+^ macrophages and epithelial cells in infected lungs at day 1 p.i. by histology^*a*^

Virus	Mean no. ± SEM of^*b*^:	
	eGFP^+^ macrophages	eGFP^+^ epithelial cell clusters
WT	176 ± 30	11 ± 0.9
ΔFCSv1	61 ± 6.6^*c*^	11 ± 1.5
ΔFCSv3	44 ± 5.0^*c*^	11 ± 1.7
ΔFCSv1 R	254 ± 31	11 ± 1.2

a BALB/c mice were infected intranasally with 10^6^ eGFP units/mouse of eGFP^+^ WT, ΔFCSv1 and ΔFCSv3 mutant, and ΔFCSv1 revertant viruses in a volume of 30 μl under general anesthesia.

b Mean number ± standard error of the mean per histological section (*n* = 6 sections). Equivalent numbers were obtained in a repeat experiment.

c The numbers of eGFP^+^ macrophages were significantly lower for the gB FCS^−^ (ΔFCSv1 and ΔFCSv3) than for the gB FCS^+^ (WT and ΔFCSv1 R) viruses (*P* < 10^−4^ by Student's *t* test).

### gB FCS^−^ viruses show subtly reduced transport to lymphoid organs.

**Fig 10:**
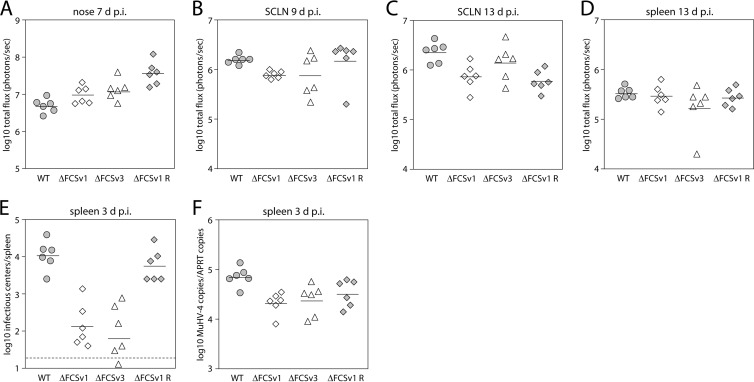
Colonization of lymphoid organs by gB FCS^−^ viruses. (A to D) Intranasal infection. BALB/c mice were infected intranasally with 10^4^ PFU/mouse of BAC^−^ WT, ΔFCSv1 and ΔFCSv3 mutant, and ΔFCSv1 revertant viruses in a volume of 30 μl under general anesthesia, and viral dissemination was monitored by *in vivo* luciferase imaging. The symbols represent single mice, while the horizontal bars show the mean value from each group. Shown are signals in the nose at day 7 p.i. (A), superficial cervical lymph nodes (SCLN) at day 9 p.i. (B), SCLN at day 13 p.i. (C), and spleen at day 13 p.i. (D). The signals in the SCLN on day 9 p.i. (B) were subtly, but significantly, lower for the gB FCS^−^ viruses (ΔFCSv1 and ΔFCSv3) than for the gB FCS^+^ viruses (WT and ΔFCSv1 R) (*P* < 0.03 by Student's *t* test). (E and F) Intraperitoneal infection. BALB/c mice were infected intraperitoneally with 10^5^ eGFP units/mouse of eGFP^+^ WT, ΔFCSv1 and ΔFCSv3 mutant, and ΔFCSv1 revertant viruses. On day 3 p.i., virus loads in spleens were determined by infectious center assay (E) and quantitative PCR (F). The splenic loads of the gB FCS^−^ viruses were significantly lower than those of the gB FCS^+^ viruses by infectious center assay (*P* < 10^−7^ by Student's *t* test) and quantitative PCR (*P* < 0.008).

Given the role of myeloid cells in trafficking virus from the site of lytic replication to lymphoid organs (19, 41), impaired myeloid infection might be expected to result in an impaired colonization of the latency reservoir. The finding that the gB FCS^−^ viruses establish normal long-term latent loads does not preclude a trafficking defect, because long-term latent loads are relatively insensitive to variation in primary lytic infection and the amount of virus seeded to the draining lymph nodes (51, 52). We therefore aimed to find out if the gB FCS^−^ viruses have a deficit in reaching lymphoid organs upon intranasal and intraperitoneal infection. The observation that the gB FCS^−^ viruses have a lytic replication deficit in the lung makes analysis of virus transport from the lung to the draining MLN difficult to interpret. In contrast, the gB FCS^−^ viruses show normal levels of lytic replication in the nose upon high-dose intranasal infection (10^4^ PFU/mouse) (Fig. 10A), rendering this site more suitable for investigating virus transport to the draining lymph nodes. While on day 9 p.i. the luciferase signals from the draining superficial cervical lymph nodes (SCLN) were slightly but significantly, lower for the gB FCS^−^ viruses than for the gB FCS^+^ viruses (Fig. 10B), no such difference was observed on day 13 p.i. (Fig. 10C). Similarly, the signals from the spleens on day 13 p.i. were not significantly different between gB FCS^−^ and gB FCS^+^ viruses (Fig. 10D). Second, we investigated the colonization of the spleen upon intraperitoneal infection, a route by which incoming virus can reach the spleen directly (53). As shown in Fig. 10E and F, the splenic loads of gB FCS^−^ viruses were significantly lower than those of gB FCS^+^ viruses as assayed by infectious center assay and quantitative PCR (qPCR) at day 3 p.i. Together, these findings suggest that the gB FCS^−^ viruses have a subtle deficit in reaching lymphoid organs upon intranasal and intraperitoneal infection.

## DISCUSSION

We investigated the role of MuHV-4 gB cleavage in host colonization by mutating its FCS. Each of three independent mutations prevented gB cleavage without affecting its expression levels, glycosylation, or antigenic conformation. The gB FCS^−^ viruses showed normal entry in BHK-21 fibroblasts and NMuMG epithelial cells but significantly reduced entry in RAW 264.7 monocytes/macrophages, *ex vivo* peritoneal macrophages, and bone marrow-derived DCs. The myeloid infection defect appeared to be postendocytic and was presumably in membrane fusion. After intranasal inoculation of mice, gB FCS^−^ viruses infected significantly fewer alveolar macrophages, while alveolar epithelial cell infection appeared normal. Thus, gB FCS loss impaired myeloid cell infection both *in vitro* and *in vivo*.

While the inefficiency of myeloid cell infection precluded a direct analysis of fusion kinetics by immunofluorescence, gB FCS^−^ viruses showed an increased susceptibility to gB-specific neutralizing antibodies blocking viral membrane fusion, consistent with a change in the fusion complex that might compromise its function. We have identified 3 gB antigenic forms: extracellular virions exclusively show one and virions after capsid and tegument release exclusively show another; these MAb-defined forms presumably correspond to pre- and postfusion gB conformations (33). MuHV-4 fuses only when it reaches late endosomes, and postendocytic, prefusion virions show a third, intermediate antigenic form of gB, which we interpret, based on analyses of the VSV glycoprotein G (6, 7), as a dynamic equilibrium between the pre- and postfusion conformations, before membrane interactions make it irreversibly postfusion (36). Normally, the MuHV-4 virion gB is bound to gH/gL (46). When gB changes from its prefusion to its intermediate form after virion endocytosis, gH/gL epitopes are also lost (36), and the gB of genetically gL^−^ virions more readily adopts the intermediate form (54), suggesting that the gB prefusion conformation is stabilized by association with gH/gL (and vice versa). gL^−^ virions also show greater susceptibility to gB-directed neutralization (47). Thus, although it was not possible to track gB conformation changes in myeloid cells, the altered neutralization profile of gB FCS^−^ mutants suggested that the normal extracellular interaction of gB with gH/gL was compromised, causing a subtle impairment of membrane fusion.

The infection defect of gB FCS^−^ MuHV-4 was consistent with that observed for similar mutants of other herpesviruses. However, cell-type-specific defects have not previously been noted. Why the requirement of MuHV-4 for gB cleavage differed between fibroblast/epithelial and myeloid infections remains unclear. It is possible that the relatively low infectivity of free MuHV-4 virions for myeloid cells—up to 100-fold less than its infectivity for fibroblast/epithelial cells—increased the impact of poor membrane fusion. This would be consistent with enhanced myeloid cell infection by bound IgG reducing the relative deficit of gB FCS^−^ viruses. Alternatively, qualitative differences that impinge on gB function may exist between fibroblast/epithelial and myeloid cell endosomes.

A significant advance here on previous data was the identification of an *in vivo* host colonization phenotype for gB FCS^−^ virus mutants. After intranasal inoculation, they showed significantly less lung infection than did WT virus at both low (10-PFU) and high (5,000-PFU) doses, with the defect being more pronounced at a low dose. There was reduced infection of alveolar macrophages, a major early target of MuHV-4 in the lung, with apparently normal alveolar epithelial cell infection. Thus, myeloid infection was selectively impaired both *in vitro* and *in vivo* by a lack of gB cleavage. Consistent with myeloid cells being important for trafficking virus from the site of lytic replication to the lymphoid organs (19, 41), the gB FCS^−^ viruses showed a subtle deficit in reaching lymphoid organs early in infection. However, from day 13 p.i. onwards, the virus loads in lymph nodes and spleens were similar for gB FCS^−^ and FCS^+^ viruses, suggesting that the onset of latency amplification leads to an equilibration of virus loads. The main *in vivo* manifestation of a lack of gB cleavage therefore seems to be reduced infection of alveolar macrophages and reduced lytic virus spread in the lung, the former presumably being responsible for the latter. As such, our *in vivo* studies demonstrated a subtle and quite specific role for gB cleavage in host colonization.
